# A Case of Pneumocystis Pneumonia Caused by Long-Term Low-Dose Steroid Therapy

**DOI:** 10.7759/cureus.93849

**Published:** 2025-10-04

**Authors:** Motoyasu Nakamura, Keisuke Suzuki, Eriko Yoshida, Kenta Watanabe, Hiroto Sasage, Ryota Takaoka, Kenji Dohi, Satoshi Suzuki

**Affiliations:** 1 General Medicine Department, Tone Health Cooperative Society, Tone Central Hospital, Gunma, JPN; 2 Department of General Medicine, Kawasaki Kyodo Hospital, Kawasaki Medical Cooperative, Kanagawa, JPN; 3 Department of Emergency, Disaster and Critical Care Medicine, Showa Medical University School of Medicine, Tokyo, JPN

**Keywords:** case report, corticosteroids, cumulative steroid dose, opportunistic infection, pneumocystis jirovecii pneumonia, rheumatoid arthritis

## Abstract

Pneumocystis pneumonia (PCP) is an opportunistic infection caused by *Pneumocystis jirovecii*. It typically occurs in patients with advanced HIV infection and severely reduced CD4+ T cell counts, but it is also seen in other immunocompromised hosts such as those receiving long-term corticosteroids, immunosuppressive agents, or chemotherapy. Common symptoms include progressive dyspnea, dry cough, and low-grade fever. In general, patients at risk for PCP who are receiving the equivalent of 20 mg/day or more of prednisolone for at least eight weeks are recommended to receive prophylactic trimethoprim-sulfamethoxazole (TMP-SMX) therapy. We report our experience of managing PCP in an 80-year-old man with a history of rheumatoid arthritis who had been receiving oral prednisolone 4 mg/day for 21 years. The patient was admitted to our hospital for rehabilitation due to disuse syndrome following surgery for aortic dissection. On hospital day one, the patient developed a fever. Oxygenation impairment was observed, prompting chest radiography and subsequent computed tomography (CT) imaging, which revealed reticular shadows and ground-glass opacities in both lung fields. These findings led to the suspicion of bacterial pneumonia and initiation of ceftriaxone therapy. By day four, his oxygen saturation dropped to 90% on room air, and laboratory tests showed a β-d-glucan level of 62.5 pg/ml. On day five, the antibiotic regimen was switched to meropenem, vancomycin, and TMP-SMX. Bronchoscopy was considered unsafe because of the patient’s general condition, and sputum polymerase chain reaction (PCR) was performed instead, which was positive for *Pneumocystis jirovecii*. Based on the PCR results, clinical course, and imaging findings, a diagnosis of PCP was made. ST and atovaquone were administered for a total of three weeks, resulting in defervescence and significant improvement in oxygenation. Even low-dose corticosteroid therapy is associated with an increased risk of opportunistic infections, depending on the cumulative dose. Although our patient had been receiving only a low daily dose, the cumulative exposure indicated a high risk for infections such as PCP. In conclusion, our case experience suggests that in patients receiving long-term corticosteroid therapy, PCP and other opportunistic infections must be considered in the differential diagnosis of pneumonia, regardless of the daily corticosteroid dose received.

## Introduction

Pneumocystis pneumoniae (PCP) is an opportunistic infection caused by *Pneumocystis jirovecii*. It can be fatal in immunocompromised patients, and its common symptoms include dyspnea, dry cough, and low-grade fever [1]. PCP has been reported to be more severe and associated with a higher mortality rate in non-HIV-infected immunocompromised patients than in HIV-infected patients [2,3]. Hence, prophylactic antibiotic therapy, such as oral trimethoprim-sulfamethoxazole (ST) combination therapy, is recommended for high-risk patients, especially those receiving oral prednisolone (PSL) equivalents of 20 mg/day or more for eight weeks or longer [4]. Here, we report a rare case of PCP caused by long-term use (for 20 years) of 4 mg PSL, a dose lower than 20 mg.

## Case presentation

The patient was a man in his 80s. His medical history included rheumatoid arthritis for over 20 years, postoperative Stanford type A aortic dissection, and benign prostatic hyperplasia. His medications comprised PSL 4 mg, vonoprazan 20 mg, and silodosin 8 mg once daily after breakfast and rebamipide 300 mg three times daily after meals.

He was admitted to our hospital for rehabilitation after surgery for acute aortic dissection, with a diagnosis of disuse syndrome. During the hospital stay, he underwent rehabilitation. The day before his referral to our department, the patient developed a fever of 38.9°C. Initially, the patient was suspected of having an upper respiratory tract infection and was managed with only antipyretic and analgesic medications under close observation. However, as there was no clinical improvement or resolution of fever on the first day, the patient was referred to our department for further evaluation and management.

The patient’s vital signs at the time of referral to our department were as follows: consciousness level, Glasgow Coma Scale (GCS) E4V5M6; blood pressure, 134/85 mmHg; heart rate, 107 beats/min; respiratory rate, 12 breaths/min; body temperature, 39.1°C; and oxygen saturation, 98% while breathing room air. Physical examination revealed normal heart sounds, with the absence of third and fourth heart sounds and no murmurs. Lung sounds were clear without adventitious sounds or left-right differences. The abdomen was flat and soft, with no abnormal bowel sounds or tenderness. Laboratory tests showed an elevated white blood cell count and C-reactive protein. Urine and blood cultures were negative. Sputum cultures revealed methicillin-sensitive *Staphylococcus aureus* (MSSA) and *Candida glabrata*, which were suspected to represent the indigenous flora (Table 1).

**Table 1:** Laboratory findings on admission WBC: white blood cell count; RBC: red blood cell count; Hb: hemoglobin; Plt: platelet count; AST: aspartate aminotransferase; ALT: alanine aminotransferase; LDH: lactate dehydrogenase; ALP: alkaline phosphatase; γ-GT: gamma-glutamyl transferase; CPK: creatinephosphokinase; BUN: blood urea nitrogen; Cre: creatinine; Na: sodium; K: potassium; Cl: chloride; CRP: C-reactive protein; β-D-glucan: beta-D-glucan; PR3-ANCA: proteinase 3-anti-neutrophil cytoplasmic antibody; MPO-ANCA: myeloperoxidase-anti-neutrophil cytoplasmic antibody; SP-A: surfactant protein A; SP-D: surfactant protein D; KL-6: Krebs von den Lungen-6; anti-DNA antibody: anti-double stranded DNA antibody; antinuclear antibody, antinuclear antibody; C. neoformans: *Cryptococcus neoformans*; *Aspergillus *antigens; HIV antigen/antibody: human immunodeficiency virus antigen/antibody.

Test Item	Result	Reference Range
WBC	9000/μL	3500-9000/μL	
RBC	4.07×10^6 /μL	4.0-5.5×10^6 /μL
Hb	12 g/dL	12.0-16.0 g/dL
Plt	25.4×10^4 /μl	15-35×10^4 /μL
AST	24 U/L	13-30 U/L	
ALT	14 U/L	8-42 U/L
LDH	218 U/L	120-245 U/L
ALP	89 U/L	38-113 U/L
γ-GT	12 U/L	10-47 U/L
CPK	30 U/L	45-163 U/L
BUN	11.6 mg/dL	8-20 mg/dL
Cre	0.52 mg/dL	0.6-1.1 mg/dL
Na	129 mEq/L	135-147 mEq/L
K	94 mEq/L	3.6-5.0 mEq/L
Cl	3.9 mEq/L	98-107 mEq/L
CRP	8.01 mg/dL	<0.3 mg/dL
β-D-glucan	62.5 pg/mL	<20 pg/mL
PR3-ANCA	<1.0 U/mL	<3.5 U/mL
MPO-ANCA	<1.0 U/mL	<3.5 U/mL
SP-A	70.4 ng/mL	<43.8 ng/mL
SP-D	36.5 ng/mL	<110 ng/mL
KL-6	414 U/L	<500 U/L
anti-DNA antibody	<2.0 IU/mL	<10 IU/mL
antinuclear antibody	<40x	<80x
C. neoformans	(-)	negative
*Aspergillus* antigens	0.3	<0.5
HIV antigen/antibody	(-)	negative
urine cell culture	(-)	negative
blood culture	(-)	negative
sputum culture	S. aureus 3+, C. Glabrata 2+	negative
*Acidophilic* bacteria culture	(-)	negative

**Figure 1:**
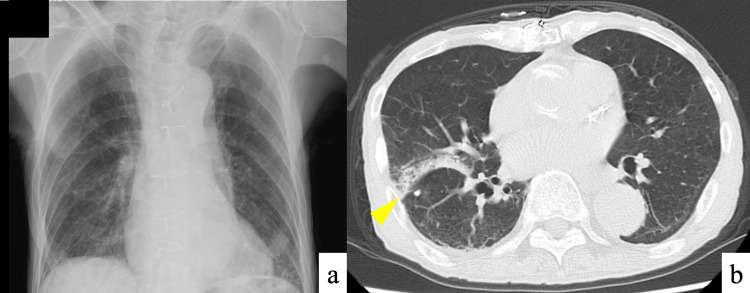
Initial chest imaging at onset of fever (a) Chest radiograph showing decreased transparency in both lower lobes. (b) Chest computed tomography (CT) without contrast demonstrating scattered ground-glass opacities in both lung fields and reticular opacities in the right lower lobe.

Imaging findings included a plain chest radiograph showing decreased opacity in both lower lobes (Figure 1a). Chest computed tomography (CT) without contrast revealed scattered ground-glass opacities in both the lung fields and reticular opacities in the right lower lobe (Figure 1b).

**Figure 2:**
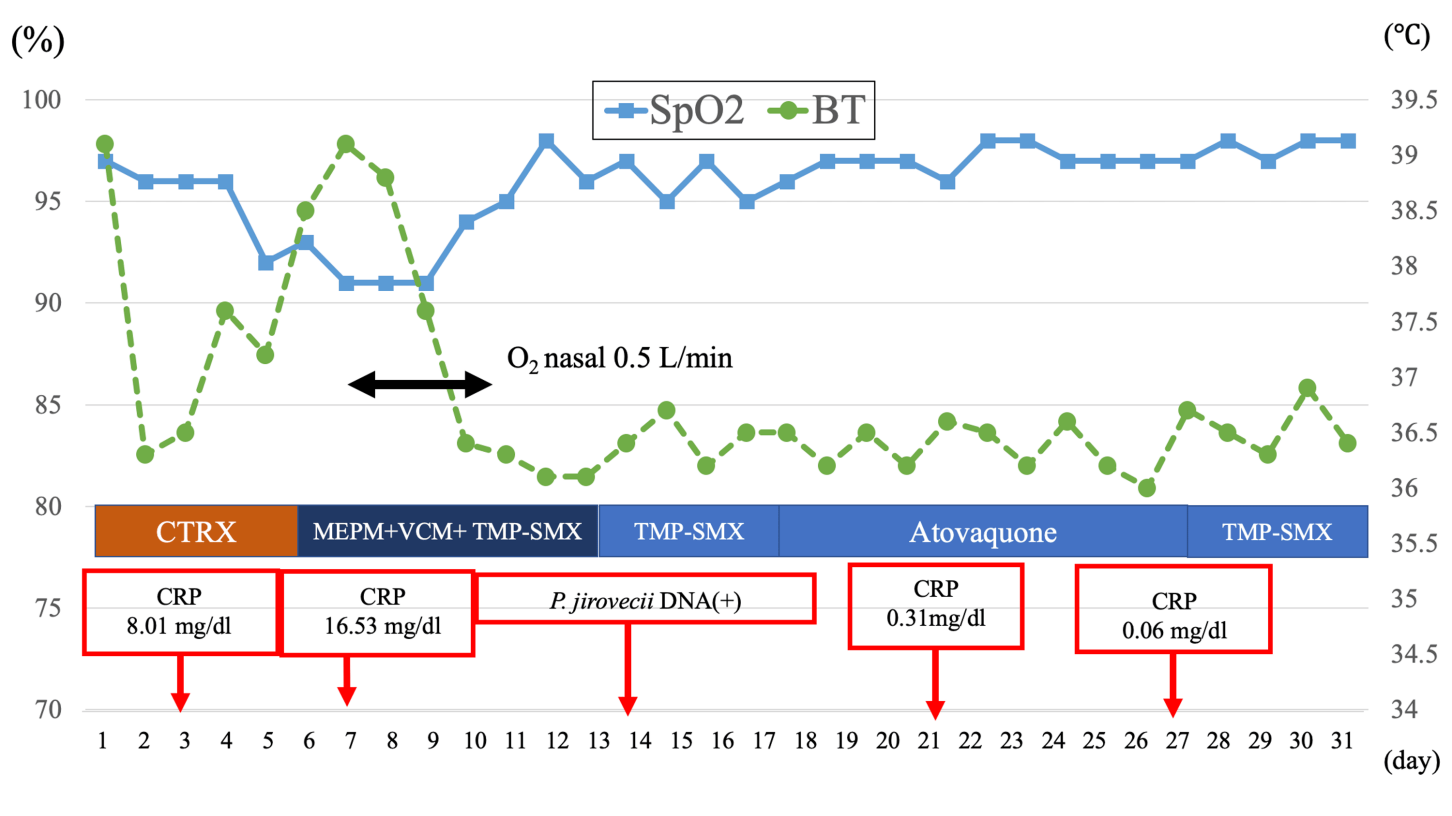
Clinical course of fever, inflammatory response, and oxygenation The image is created by the author. The patient’s temperature, oxygen saturation, and inflammatory parameters showed improvement after initiation of trimethoprim-sulfamethoxazole (TMP-SMX) followed by atovaquone.

Based on imaging findings and a mild inflammatory response, the patient was diagnosed with bacterial pneumonia, and ceftriaxone (CTRX) 2 g every 24 h was initiated on day one. After the initiation of CTRX treatment, the fever subsided on day two. However, his oxygen saturation gradually decreased, and the fever recurred, reaching 38°C on day four. A repeat chest CT performed on day five revealed worsening ground-glass opacities (Figure 2).

The antibiotic regimen was then changed to meropenem (MEPM) and vancomycin (VCM). Because bronchoalveolar lavage (BAL) was judged unsafe owing to the patient’s general condition, polymerase chain reaction (PCR) testing for Pneumocystis jirovecii was performed using a sputum sample. Meanwhile, trimethoprim 320 mg and sulfamethoxazole 1,600 mg were started because of elevated β-d-glucan levels and the clinical suspicion of *Pneumocystis pneumonia* (PCP). Although the patient’s respiratory failure was assessed as moderate, corticosteroids were not initially administered as a treatment for presumptive PCP because a definitive diagnosis was lacking. Instead, adrenal insufficiency was suspected, and PSL (20 mg) was administered on days 5 to 8. Because the patient’s general condition improved, concomitant corticosteroid therapy specifically targeting the PCP was not recommended.

**Figure 3:**
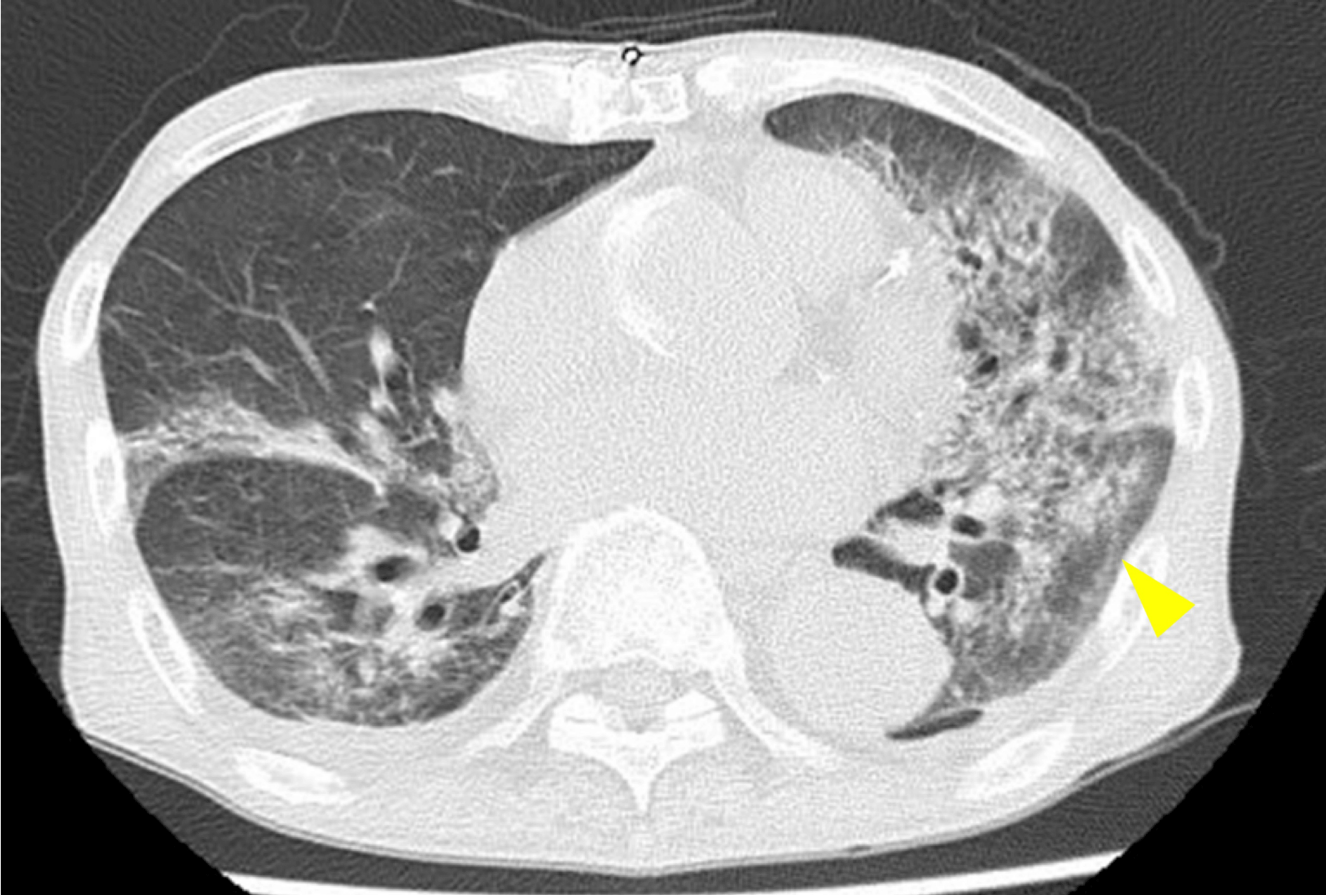
Follow-up chest CT on hospital day five Chest CT demonstrating progression of bilateral ground-glass opacities compared with initial imaging, despite antibiotic therapy for suspected bacterial pneumonia.

By hospital day eight, the fever subsided and oxygenation improved (Figure 3).

On day 13, PCR analysis of a sputum sample collected on day five was positive for *Pneumocystis jirovecii* DNA. Considering the clinical course under ST treatment, a definitive diagnosis of PCP was established. However, on day 17, the patient developed severe nausea, which led to a switch in therapy to atovaquone 750 mg.

**Figure 4:**
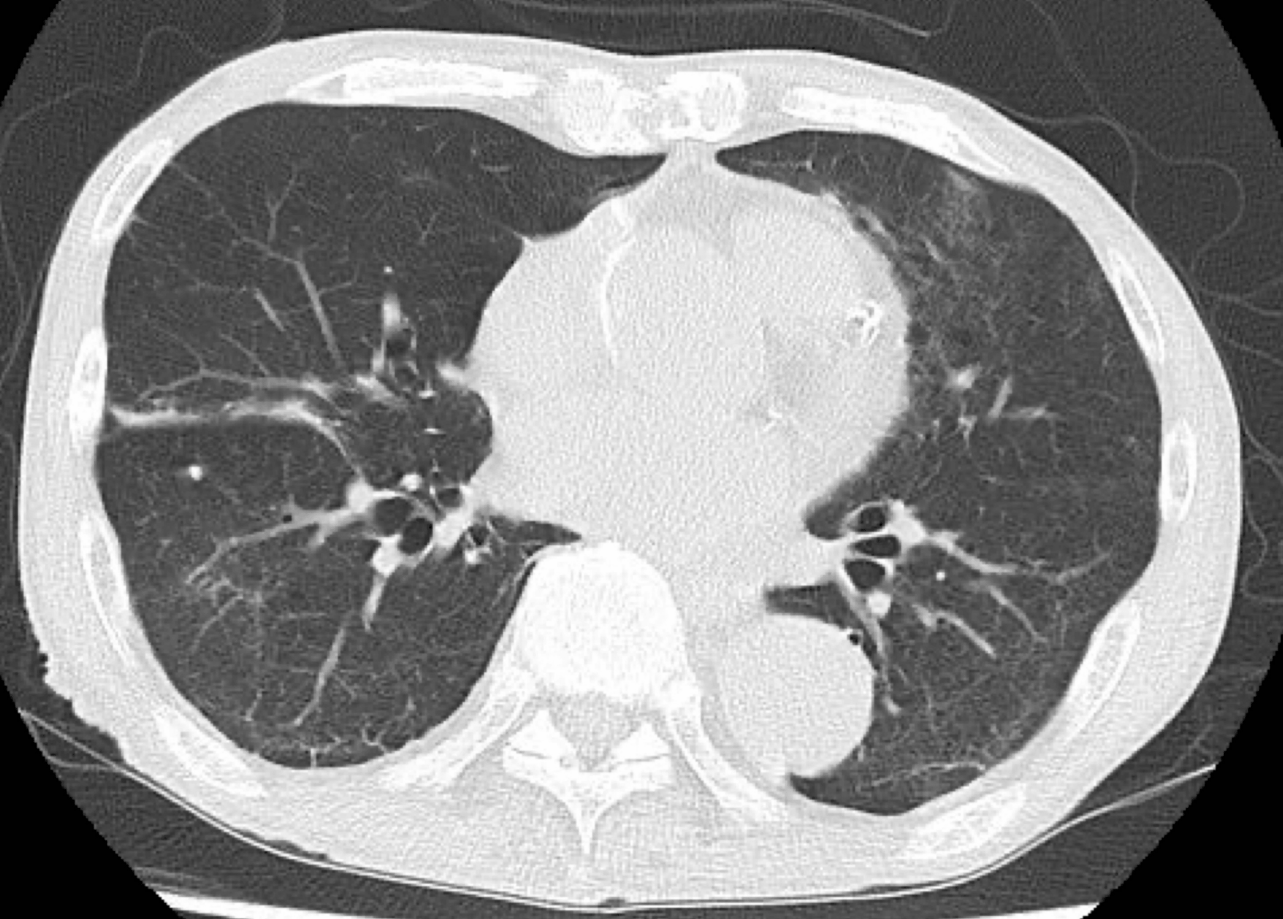
Follow-up chest CT on hospital day 27 Chest CT showing resolution of bilateral ground-glass opacities following completion of three weeks of treatment with trimethoprim-sulfamethoxazole and atovaquone.

The treatment continued for 21 days. Follow-up chest CT on day 27 showed that the ground-glass opacities had disappeared (Figure 4).

The patient was subsequently transitioned to prophylactic oral trimethoprim 80 mg plus sulfamethoxazole 400 mg and was discharged to a rehabilitation facility on hospital day 31.

## Discussion

We describe our experience of managing a patient who developed PCP while being on a long-term low-dose steroid (4 mg) treatment for rheumatoid arthritis. The patient’s condition improved after ST and atovaquone treatments.

The most important risk factor for PCP in non-HIV-infected patients is the use of immunosuppressants, particularly corticosteroids [5]. The incidence of opportunistic infections increases not only with doses of 20 mg/day or more in PSL equivalents but also with cumulative doses. Specifically, cumulative PSL doses of 700 mg or more, or even low doses of less than 10 mg/day in PSL equivalents, can increase the risk of developing general infections [6]. Furthermore, those taking 5 mg PSL for three months, six months, and three years, respectively, have a 30%, 46%, and 100% increased risk of serious infections, compared with non-users. In particular, the risk of taking 5 mg PSL for three years has been reported to be similar to that associated with a 30 mg dose taken for one month [7].

Although the dose in this case was low, the eventual cumulative dose indicated a high risk of opportunistic infections. This cumulative dose would be reached in approximately six months with a daily dose of 4 mg. The patient had been receiving steroids for more than 20 years; therefore, the cumulative dose exceeded 700 mg. It is believed that the cumulative dose, resulting from long-term oral administration of PSL, increased the risk of PCP and led to its development. Furthermore, because the patient developed PCP and required continued oral administration of low-dose PSL (4 mg), we decided to administer prophylactic antibiotic treatment with ST. However, in a 14-year long-term (four weeks or more) study of non-high-dose steroids (low doses based on prednisone [<15 mg/day]), the one-year PCP incidence rate in the low-dose group was 0.01 (95% CI: 0.001-0.03)/100 person-years, and it remains unclear whether prophylactic antibiotic treatment is justified in patients receiving low-dose glucocorticoid treatment at <15 mg/day [8]. Therefore, future research is needed regarding the need for prophylactic antibiotic treatment in patients receiving low-dose PSL at doses <15 mg/day. However, in this case, considering the patient's history of PCP, oral prophylaxis was considered necessary. This case showed that even low-dose oral steroid use can increase the risk of developing PCP. When pneumonia develops in patients receiving long-term low-dose PSL, risk assessment, considering the previous duration and dosage, and differentiation from PCP are necessary. Recognizing the risk of PCP when diagnosing pneumonia in patients receiving low-dose oral steroids may enable earlier diagnosis and treatment, leading to improved patient outcomes.

This was a single-case report; therefore, the generalizability of our findings is limited. The diagnosis of PCP was based on the clinical course, as no evaluations, such as BAL, were performed. Furthermore, given that this patient was older (in his 80s) and had been diagnosed with disuse syndrome, a decline in activities of daily living cannot be completely ruled out as a possible cause of the opportunistic infection. Finally, the need for oral treatment to prevent PCP in patients receiving long-term low-dose oral steroids requires further comparative studies to assess the risk of onset and to evaluate patients who require oral steroids.

## Conclusions

This report presents a rare case of PCP in a patient with rheumatoid arthritis who had been receiving low-dose (4 mg/day) PSL for a long time. Although PCP has traditionally been observed in high-dose steroid users, our findings suggest that even in patients receiving low-dose steroids, the risk of infection may increase significantly with increasing duration of use and cumulative dose. In the future, it will be necessary to assess the risk of PCP in patients receiving low-dose steroids, taking into account their medical history, duration of steroid use, and cumulative steroid dose.

## References

[1] Bateman M, Oladele R, Kolls JK (2020). Diagnosing Pneumocystis jirovecii pneumonia: a review of current methods and novel approaches. Med Mycol.

[2] Mansharamani NG, Garland R, Delaney D, Koziel H (2000). Management and outcome patterns for adult Pneumocystis carinii pneumonia, 1985 to 1995: comparison of HIV-associated cases to other immunocompromised states. Chest.

[3] Sepkowitz KA (2002). Opportunistic infections in patients with and patients without acquired immunodeficiency syndrome. Clin Infect Dis.

[4] Yale SH, Limper AH (1996). Pneumocystis carinii pneumonia in patients without acquired immunodeficiency syndrome: associated illnesses and prior corticosteroid therapy. Mayo Clin Proc.

[5] Park JW, Curtis JR, Moon J (2018). Prophylactic effect of trimethoprim-sulfamethoxazole for pneumocystis pneumonia in patients with rheumatic diseases exposed to prolonged high-dose glucocorticoids. Ann Rheum Dis.

[6] Stuck AE, Minder CE, Frey FJ (1989). Risk of infectious complications in patients taking glucocorticosteroids. Rev Infect Dis.

[7] Dixon WG, Abrahamowicz M, Beauchamp ME (2012). Immediate and delayed impact of oral glucocorticoid therapy on risk of serious infection in older patients with rheumatoid arthritis: a nested case-control analysis. Ann Rheum Dis.

[8] Park JW, Curtis JR, Kim MJ (2019). Pneumocystis pneumonia in patients with rheumatic diseases receiving prolonged, non-high-dose steroids-clinical implication of primary prophylaxis using trimethoprim-sulfamethoxazole. Arthritis Res Ther.

